# Genogroup I and II Picobirnaviruses in Respiratory Tracts of Pigs

**DOI:** 10.3201/eid1712.110934

**Published:** 2011-12

**Authors:** Saskia L. Smits, Leo L.M. Poon, Marije van Leeuwen, Pui-Ngan Lau, Harsha K.K. Perera, Joseph S. Malik Peiris, James H. Simon, Albert D.M.E. Osterhaus

**Affiliations:** Erasmus Medical Center, Rotterdam, the Netherlands (S.L. Smits, A.D.M.E. Osterhaus);; The University of Hong Kong, Hong Kong, People’s Republic of China (L.L.M. Poon, P.-N. Lau, H.K.K. Perera, J.S. Malik Peiris);; Viroclinics Biosciences BV, Rotterdam (S.L. Smits, M. van Leeuwen, J.H. Simon, A.D.M.E. Osterhaus);; University of Kelaniya, Kelaniya, Sri Lanka (H.K.K. Perera)

**Keywords:** pigs, viruses, picobirnavirus, porcine picobirnavirus, genogroup I, genogroup II, respiratory tract, phylogeny, random amplification, slaughterhouses, zoonoses, Hong Kong, Sri Lanka

## Abstract

Sequence-independent amplification and specific reverse transcription PCRs identified genogroup I and II picobirnaviruses in respiratory tracts of pigs. These data expand knowledge of picobirnavirus diversity and tropism. Genetic relationships between porcine respiratory and human enteric picobirnaviruses suggest cross-species transmission of picobirnaviruses between pigs and humans.

A thorough understanding of virus diversity in animals provides epidemiologic baseline information about potential pathogens and can lead to identification of emerging human pathogens. On the basis of relevance to reemerging viruses, such as Nipah virus and influenza A virus, pigs are a key risk host for emerging RNA virus–associated disease in humans in different areas (*1**–**3*). In an effort to identify unknown porcine viruses in the respiratory tracts of pigs, we performed sequence-independent amplification on partially purified viral nucleic acid from swab samples of respiratory tracts from pigs that were PCR negative for influenza A virus (*4**–**6*).

## The Study

We analyzed 197 respiratory tract swab specimens from pigs obtained in slaughterhouses in Hong Kong, China, and Colombo, Sri Lanka. Large-scale molecular RNA virus screening, based on host nucleic acid depletion, sequence-independent amplification, and sequencing of partially purified viral RNA was performed on nucleic acids isolated from 10 respiratory tract swab samples from pigs (*4**–**6*). Most of the 893 analyzed sequences were of unclassified porcine genome origin, unclassified, or bacterial origin. Three clones showed picobirnaviruses, which are double-stranded RNA viruses with a segmented genome belonging to the family *Picobirnaviridae* (*7*).

Because picobirnaviruses have been identified only in fecal specimens, diagnostic PCRs for genogroup I and II picobirnaviruses specific for the RNA-dependent RNA polymerase (RdRp) gene (*6**,**8**,**9*) were performed on 60 respiratory tract swab specimens to determine whether picobirnaviruses are present in the porcine respiratory tract. Sixteen (26.6%) of 60 samples were confirmed by sequencing as positive for genogroup I picobirnaviruses, and 4 (6.5%) of 60 were confirmed by sequencing as positive for genogroup II picobirnaviruses. Three of 60 porcine swab samples that showed evidence of genogroup II picobirnaviruses were also positive for genogroup I picobirnavirus.

Subsequent sequence analysis suggested that the genogroup II pan-picobirnavirus PCR might be suboptimal for detecting the novel genogroup II swine picobirnaviruses. Thus, we designed a new genogroup II diagnostic PCR that used primers 5′-GACCGGTWTGGATGTTTCCGATG-3′ and 5′-GTATCTGTGCTGGSCGCAT-3′, AmpliTaq Gold DNA polymerase (Life Technologies, Carlsbad, CA, USA), and 2 mmol/L MgCl_2_ (incubation at 95°C for 10 min, followed by 40 cycles at 95°C for 30 s, 56°C for 30 s, and 72°C for 20 s), and screened an additional 137 porcine respiratory tract swab samples for genogroup II picobirnaviruses. Twenty-two (16.0%) samples were positive for genogroup II picobirnaviruses.

**Figure A1:**
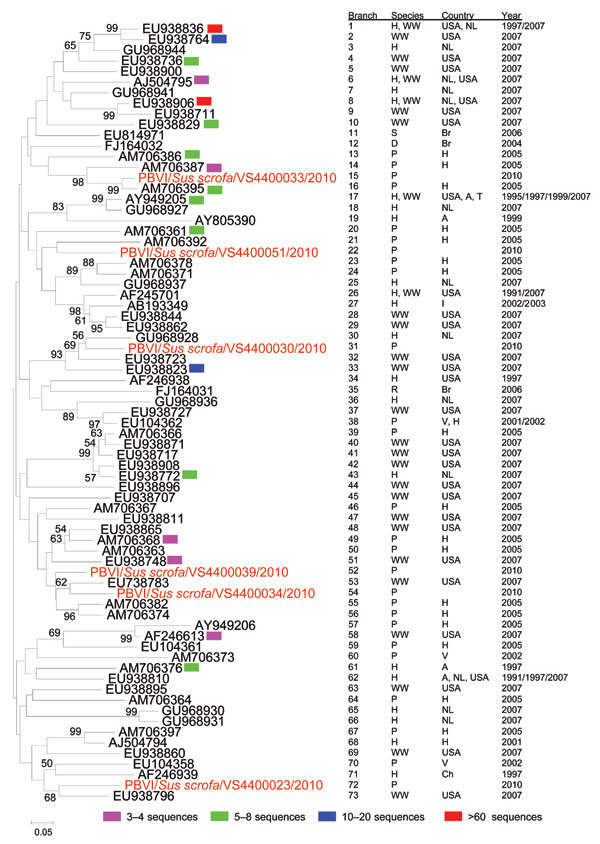
Neighbor-joining (Jukes-Cantor model) phylogenetic tree of an ≈165-bp fragment of the genogroup I picobirnavirus RNA-dependent RNA polymerase gene from known human, porcine, and wastewater genogroup I picobirnaviruses and newly characterized porcine respiratory genogroup I picobirnaviruses (sequences are available on request). Each branch represents a sequence or group of sequences (95% identical with gaps) depending upon the presence of a colored block. Every branch corresponds to the adjacent information, which indicates source, year of isolation, and location of virus variants. Reference sequences from published studies are identified by their GenBank accession numbers. For species, H, human; WW, wastewater; S, snake; D, dog; P; pig; R, rat. For countries of isolation, USA, United States; NL, the Netherlands; Br, Brazil; H, Hungary; A, Argentina; T, Thailand; In, India; V, Venezuela; Ch, People’s Republic of China. Sequences from newly identified porcine respiratory picobirnaviruses in this study are shown according to recent nomenclature proposals (*7*). Isolates in red are branches in which porcine respiratory picobirnaviruses are present. Scale bar indicates nucleotide substitutions per site.

To determine genetic relationships between porcine respiratory genogroup I picobirnaviruses with reported genogroup I viruses detected in wastewater and human and porcine feces, a phylogenetic tree was constructed on the basis of a 165-nt fragment of the RdRP gene as described (*6**,**10*) (Figure A1). Genogroup I picobirnavirus nucleotide sequences from 6 porcine respiratory samples showed 58%–80% similarity with each other and belonged to different phylogenetic clades.

PBVI/*Sus scrofa*/VS4400033/2010 and PBVI/*Sus* *scrofa*/VS4400051/2010 were most closely related to porcine enteric picobirnaviruses identified in Hungary in 2005. PBVI/*Sus* *scrofa*/VS4400034/2010, PBVI/*Sus scrofa*/VS4400039/2010, and PBVI/*Sus scrofa*/VS4400023/2010 clustered with picobirnaviruses identified in wastewater in the United States in 2007. PBVI/*Sus scrofa*/VS4400030/2010 was most closely related to an enteric human picobirnavirus identified in the Netherlands in 2007. The close relationship between human and porcine genogroup I picobirnaviruses can also be observed in other parts of the phylogenetic tree, e.g., in branches 23–25 and 70–71 (Figure A1).

To determine genetic relationships between porcine respiratory genogroup II picobirnaviruses with reported genogroup II viruses detected in human feces, a phylogenetic tree was constructed on the basis of a 339-nt fragment of the RdRP gene as described (*6**,**10*) (Figure). Porcine genogroup II picobirnavirus partial RdRP nucleotide sequences from samples VS4400017, VS4400028, VS4400041, and VS4400049 showed 69%–96% similarity with each other (Table). Genogroup II picobirnaviruses PBVII/*Sus scrofa*/VS4400017/2010 and PBVII/*Sus scrofa*/VS4400049/2010 grouped in the same phylogenetic clade as human picobirnavirus genogroup II strains PBVII/*Homo sapiens*/USA-4-GA-91/1991 (*9*) and PBVII/*Homo sapiens*/NLD-142–3/2007 (*6*) and displayed only 2%–5% sequence diversity with each other, suggesting they could be considered the same virus (Figure; Table).

**Table:** Pairwise nucleotide sequence identity between genogroup II picobirnaviruses for a 339-bp fragment of the RdRP coding region*

Strain	4-GA-91 (AF246940)	VS142–3 (GU968925)	GPBV6G2P (AB526257)	VS4400017	VS4400028	VS4400041	VS4400049
4-GA-91 (AF246940)		97.1	68.8	95.3	70.5	69.6	97.9
VS142–3 (GU968925)			68.5	95.3	69.3	69.3	97.9
GPBV6G2P (AB526257)				67.9	79.5	75	68.2
VS4400017					69.9	69	95.6
VS4400028						74.7	69.9
VS4400041							68.8
VS4400049							

*Reference strain 4-GA-91, GenBank accession no. AF246940. Values are percentages. Blank spaces indicate 100% identity. RdRP, RNA-dependent RNA polymerase.

Genogroup II picobirnaviruses PBVII/*Sus scrofa*/VS4400028/2010 and PBVII/*Sus* *scrofa*/VS4400041/2010 could be grouped with human picobirnavirus genogroup II strain PBVII/*Homo sapiens*/IND-GPBV6G2P/2007 from India but displayed 20%–30% sequence diversity with each other (Figure; Table). Close genetic relationships could also be observed between genogroup II picobirnaviruses from pigs and humans.

## Conclusions

Our results indicated that picobirnaviruses can be commonly found in the respiratory tract of pigs from different locations and identified genogroup II picobirnaviruses in animals. Picobirnaviruses have been regarded as enteric viruses because all described cases were associated with virus shed in feces. Picobirnaviruses have been detected in human patients with and without gastroenteritis and are found in patients co-infected with enteric pathogens such as rotaviruses, caliciviruses, and astroviruses (*8**,**9*). Prevalence studies of picobirnaviruses in immunocompromised patients suggest that picobirnaviruses might be opportunistic enteric pathogens (*11**,**12*). Picobirnaviruses in the respiratory tracts of pigs suggest that picobirnaviruses might be not only potential enteric pathogens but also respiratory pathogens. The pigs used in our study showed no evidence of overt respiratory or other disease at the time of sampling. Whether these viruses contribute to disease early in life remains unclear.

Genogroup II porcine picobirnaviruses detected in this study were highly diverse. Phylogenetic analysis of porcine and human genogroup II picobirnaviruses indicated that >2 phylogenetic clades of genogroup II picobirnaviruses co-exist in these populations. Similar observations were found regarding the high genetic diversity of porcine respiratory genogroup I picobirnaviruses. Genetic relationships between porcine respiratory picobirnaviruses and picobirnaviruses from wastewater in the United States and human feces were observed. These results suggest that there were multiple cross-species transmissions of picobirnavirus strains between swine and humans (*8**–**10**,**13**–**15*).

Because molecular characterization of picobirnaviruses is limited, mostly to partial RdRP sequences, and only 1 complete genome has been determined, the zoonotic potential of picobirnaviruses awaits further characterization of full-length genomes. Attempts to obtain full-length genomes in this study were undertaken by using next-generation sequencing platforms but were unsuccessful. However, the extent of sequence variation along the 165-bp fragments of the RdRP gene of genogroup I picobirnaviruses for which an entire RdRP gene sequence is available (strains 1-CHN-97, HY005102, and VS10, and GenBank accession nos. AB186898, AF246939, and GU968924) show good correlation with overall sequence variation observed in the entire RdRP gene (*13*).

A thorough understanding of the diversity of viruses in animals, virus transmission routes, and virus tropism provides epidemiologic baseline information about potential pathogenic threats from animal reservoirs for human health. Detection of genogroup I and II picobirnaviruses in porcine respiratory tract swab samples is an example of the needed expansion of our knowledge of picobirnavirus diversity and also expands our knowledge of picobirnavirus tropism. To better understand the epidemiology of genogroup II picobirnaviruses in pigs and to define whether zoonotic or reverse-zoonotic transmissions occur, more intensive surveillance on this group of virus in pigs from other regions needs to be conducted. Whether genogroup I and/or II picobirnaviruses can also be detected in the human respiratory tract and whether they play a causal role in respiratory diseases remain to be determined. This study illustrates how novel molecular techniques can provide new understanding of viral ecology, evolution, and spread.

## Figures and Tables

**Figure:**
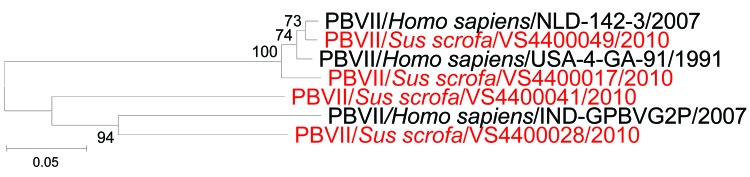
Phylogenetic analysis of genogroup II picobirnaviruses. Neighbor-joining (Jukes-Cantor model) phylogenetic tree of an ≈339-bp fragment (reference strain 4-GA-91 (*9*) of the picobirnavirus genogroup II RNA-dependent RNA polymerase gene from known human picobirnaviruses and newly characterized porcine picobirnaviruses in this study (JN176312–315, shown in red). Significant bootstrap values are shown. Nomenclature of depicted viruses is according to recent proposals (*7*). PBVII/*Homo sapiens*/USA-4-GA-91/1991 (AF246940) (*9*); PBVII/*Homo sapiens*/NLD-VS142–3/2007 (GU968925) (*6*); and PBVII/*Homo sapiens*/IND-GPBV6G2P/2007 (AB526257). Scale bar indicates nucleotide substitutions per site.
